# Intelligent Tire Sensor-Based Real-Time Road Surface Classification Using an Artificial Neural Network

**DOI:** 10.3390/s21093233

**Published:** 2021-05-07

**Authors:** Dongwook Lee, Ji-Chul Kim, Mingeuk Kim, Hanmin Lee

**Affiliations:** Department of Smart Industrial Machine Technologies, Korea Institute of Machinery and Materials, 156 Gajeongbuk-Ro, Yuseong-Gu, Daejeon 34103, Korea; lego0410@kimm.re.kr (D.L.); jckim@kimm.re.kr (J.-C.K.); Kmg0702@kimm.re.kr (M.K.)

**Keywords:** intelligent tire, road surface classification, deep neural network

## Abstract

Vehicles today have many advanced driver assistance control systems that improve vehicle safety and comfort. With the development of more sophisticated vehicle electronic control and autonomous driving technology, the need and effort to estimate road surface conditions is increasing. In this paper, a real-time road surface classification algorithm, based on a deep neural network, is developed using a database collected through an intelligent tire sensor system with a three-axis accelerometer installed inside the tire. Two representative types of network, fully connected neural network (FCNN) and convolutional neural network (CNN), are learned with each of the three-axis acceleration sensor signals, and their performances were compared to obtain an optimal learning network result. The learning results show that the road surface type can be classified in real-time with sufficient accuracy when the longitudinal and vertical axis acceleration signals are trained with the CNN. In order to improve classification accuracy, a CNN with multiple input that can simultaneously learn 2-axis or 3-axis acceleration signals is suggested. In addition, by analyzing how the accuracy of the network is affected by number of classes and length of input data, which is related to delay of classification, the appropriate network can be selected according to the application. The proposed real-time road surface classification algorithm is expected to be utilized with various vehicle electronic control systems and makes a contribution to improving vehicle performance.

## 1. Introduction

The recent advancement of vehicle control technology has resulted in several different driving assistance systems that offer convenience and safety to drivers [1,2,3]. However, automotive companies are still competing to release vehicles equipped with better advanced driver assistance systems (ADAS), and research is ongoing to improve the reliability of vehicle electronic control systems for autonomous driving technology that can replace human drivers. More vehicle and environment information must be collected to enhance vehicle control systems; for a long time, researchers have attempted to estimate road surface conditions or potential tire grip because road surface condition is one of the key factors in improving the stability and performance of vehicle control [4].

Road surface condition information can be used to improve driver safety and comfort in a variety of ways [5,6,7,8]. Reliable road surface information could directly improve many electronic vehicle control systems—such as electronic stability control (ESC), adaptive cruise control (ACC) and autonomous emergency braking (AEB)—because it can be used to estimate the maximum road friction coefficient, which indicates how much of the contact force between the tires and the road can be utilized for accelerating, braking and steering the vehicle. In addition, for autonomous driving, road surface condition information is essential for adjusting the threshold of vehicle control input, such as engine throttle, brake and steering, to prevent sliding or locking in rainy or icy road conditions. Moreover, it is possible to improve the convenience by utilizing the roughness of the road to control an active suspension system that changes the suspension properties.

Many previous studies have been conducted to estimate road surface conditions or the road friction coefficient. The methods used in these studies can be divided into three categories: (1) tire force-slip-based methods, (2) road property-based methods, and (3) tire–road interaction-based methods.

The tire force-slip-based method has been applied most often in previous studies. Based on a vehicle and tire dynamics model, the behavior of the vehicle and tires are measured, the tire contact force and slip ratio (the speed ratio between the driving and driven wheels) are estimated, and the resulting force-slip relationship is used to determine the potential grip of the vehicle on the road [9,10,11,12,13]. This approach has the advantage of making it possible to estimate the road surface friction coefficient with numerical value from the vehicle dynamics model, and the number of experiments required is relatively small because the experimental results are needed only for identifying the parameters of a physical model. However, due to the difficulty in estimating the exact parameters of the vehicle model, and in applying tire force-slip-based approaches in low excitation situations, these approaches have limitations for application prospects in general driving situations.

A road property-based method classifies the road surface by extracting the physical properties of the road surface by using noncontact sensors, such as cameras, light detection and ranging (LiDAR), radar, ultrasonic sensors, or optical sensors [14,15,16,17,18]. This approach has been applied mainly in autonomous vehicles that are basically installed with noncontact sensors including cameras and LiDAR. These methods have the advantage of being able to operate even when the vehicle is not driving; however, the performance of road property-based methods can degrade significantly in adverse weather conditions such as snow, rain, fog and sunglare. Moreover, the camera image-based approach requires a large database of experiments that reflect a variety of environmental conditions, including changes in region, weather conditions and the intensity of illumination.

In tire–road interaction-based methods, road surface conditions are classified by measuring tire vibration or deformation caused by the friction force on the tire contact patch [19,20,21,22,23]. Similar to road property-based methods, tire–road interaction-based methods have seen an upswing in research activity in recent years because these approaches can obtain useful signals that reflect road surface properties even in low-excitation situations. Tire–road interaction-based methods also offer the additional benefit that most of the sensors these methods require are inexpensive and are more robust to the external environmental conditions than the sensors used in road property-based methods.

Among the various types of sensors that can measure tire–road interactions, intelligent tire sensor that directly measures the acceleration or deformation of tire elements through sensors installed inside the tire have been applied in many recent studies [24,25,26,27,28]. Current studies that use intelligent tires for estimating road conditions have succeeded in finding the characteristic features from the waveforms of acceleration sensor signals for different types of road surfaces through experimental data, but no algorithm that can classify road surface types from sensor signals in real time has yet been suggested. Therefore, the goal of this study is to develop an algorithm that estimates road surface type in real time by using a deep neural network (DNN) based on signals from an intelligent tire system with an accelerometer attached inside the tires.

Section 2 describes the experimental setup, data acquisition experiment using intelligent tire sensor on various road surfaces for an acceleration signal database, and the data preprocessing and training process with DNNs. In Section 3, the performances of deep learning with two different types of neural networks and with three types of acceleration signal databases (x-, y- and z-axis) are compared. In order to improve classification accuracy, a network with multiple input that can simultaneously learn 2-axis or 3-axis acceleration signals is proposed. In addition, how the accuracy is affected by the number of classes and length of input data is analyzed so that the network can be selected according to the application. By applying the trained network to actual intelligent tire sensor experimental data, the classification performance in real time was also confirmed. In Section 4, we summarize the findings of this study and provide suggestions for future work.

## 2. Materials and Methods

### 2.1. Experimental Setup

**Figure 1:**
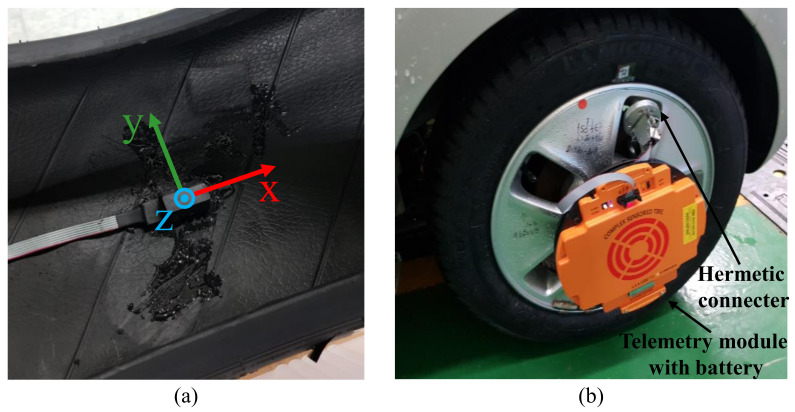
Intelligent tire system setup: (**a**) Attachment of accelerometer inside the tire. (**b**) Hermetic connector and telemetry installation on the vehicle wheel.

To measure the response to tire–road surface interactions during the contact period under various road conditions, an intelligent tire system with a three-axis accelerometer capable of measuring up to ±500 g was installed inside the tire, as shown in Figure 1a. A silylated urethane adhesive was utilized to attach the sensor inside the tire. The hermetically sealed electric connecter allows to send the signals and to receive power between the sensor inside of the high-pressurized tire and the telemetry device attached outside of the tire. The telemetry device transmits the sensor signal at a sampling rate of 1 kHz through Bluetooth wireless communication to the data acquisition(DAQ) system. Figure 1b shows the results of installing an intelligent tire system (including the accelerometer, hermetic connecter, and telemetry) on the tire and wheel of a Kia Ray test vehicle.

To collect the vehicle driving status information, the steering angle, wheel speed, and brake on/off signals were acquired from the vehicle internal controller area network (CAN) at a sampling rate of 100 Hz in synchronization with the intelligent tire sensor signal. Since the vehicle internal CAN signal was used, additional sensors other than the intelligent tire sensor were not utilized. To receive signals from intelligent tire sensors and vehicle CAN network simultaneously with time synchronization, DAQ system composed of cRIO-9063 + NI-9853 of National Instrument was utilized.

### 2.2. Data Acquisition Experiment

**Figure 2:**
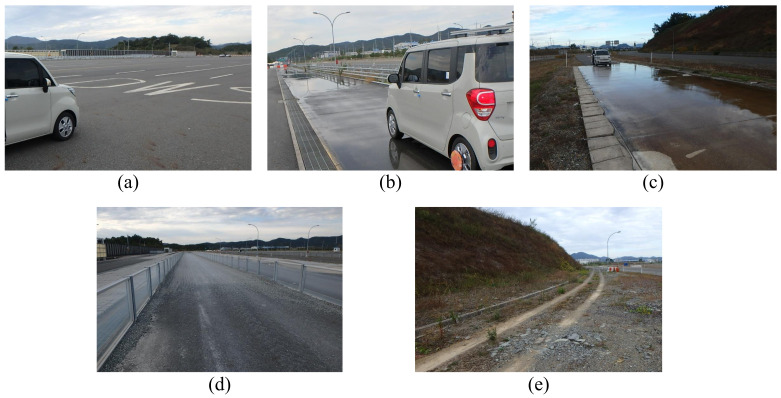
Data acquisition experiment environment of (**a**) dry asphalt, (**b**) wet asphalt with 1 mm waterfilm, (**c**) wet asphalt with 4 mm waterfilm, (**d**) gravel, and (**e**) unpaved road.

To collect an intelligent tire sensor signal database for deep learning, driving experiments were conducted on five different road surface conditions (dry asphalt, wet asphalt with a 1 mm waterfilm height, wet asphalt with a 4 mm waterfilm height, gravel, and unpaved road) at the proving ground (PG) of the Korea Intelligent Automotive Part Promotion Institute (KIAPI). Figure 2 shows the experimental environment for each road surface. Dry asphalt test road has a length of 400 m for straight test driving, and in the case of other road conditions, they have a length that allows a straight driving test of at least 100 m.

For each road condition, the driving experiment was repeated at various vehicle speeds (10, 20, 30, 40, 50, 60, and 70 kph). The vehicle was driven in a straight line while keeping the vehicle speed as constant as possible without applying the brake. It was impossible to drive at a perfectly constant vehicle speed because a person gave the driving command, but the driving test was conducted with an error of less than 10 kph from the target vehicle speed. Due to operational limitations on the gravel and unpaved roads, the experiments on these surfaces were conducted at up to only 50 and 20 kph, respectively. For each road surface and vehicle speed, the experiment was repeated until sufficient experimental data with a total effective driving distance greater than 800 m, which corresponds to more than 400 instances of contact of the intelligent tire sensor with the road surface.

**Figure A1:**
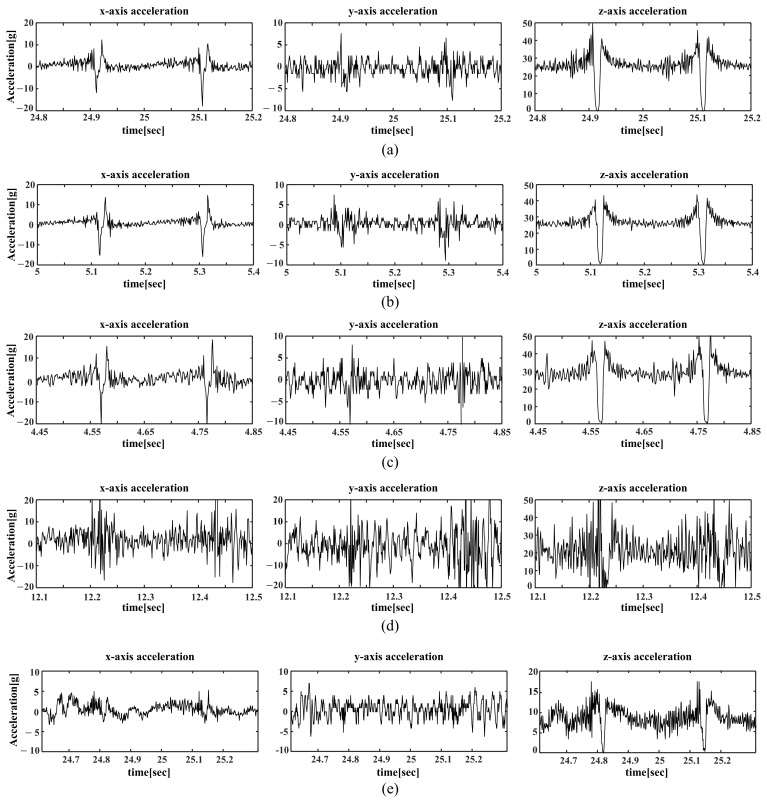
x-, y-and z-axis accelerometer measurement results measured for 30 kph experiment (20 kph for unpaved roads) at (**a**) dry asphalt, (**b**) wet asphalt with 1 mm waterfilm, (**c**) wet asphalt with 1 mm waterfilm, (**d**) gravel, and (**e**) unpaved road.

Figure A1 shows the x-, y- and z-axis accelerometer measurement results of the 30 kph experiment (20 kph for the unpaved roads), and the characteristics of the acceleration signals from each road surface type can be compared with each other. To easily compare the acceleration signals for each road surface, a time band with vehicle speed as close as 30 kph was selected to reduce the effect of vehicle speed on the signal amplitude and frequency. In the case of the unpaved road, since the experiment was performed up to only 20 kph, the signals of the 20 kph experiment were compared. The comparative analysis will be described with the learning result in the next section.

### 2.3. Data Preprocessing for Deep Learning

To refine the learning data from the raw experimental data, an effective learning data period is defined as the period with conditions in which the vehicle speed is within an error of ±10 kph from the target speed, the steering angle is under 15 degrees and the brake is in the brake-off condition. The reason for defining the effective learning data period is to train the network with the signals acquired from the tire condition close to free-rolling state without large slip. From the effective learning data period, three-axis acceleration data for deep learning were collected with a time window $\left[t_{k}\;\;\;t_{k+n-1}\right]$ with a length of *n* moving with constant intervals. The length of time window *n* is the same as the length of sequential data input to the network, and each data cut by the time window becomes one data for training, verifying or testing. Since the three-axis acceleration signal cropped by the time window has different magnitudes depending on the vehicle speed, the average and standard deviation are normalized to 0 and 1, respectively, so that the network excludes the effect by magnitude as much as possible and can learn only the waveform characteristics related to the road condition. Equation (1) shows how x-axis acceleration signal data sequence $\left[A_{x}\left(t_{k}\right)\;A_{x}\left(t_{k+1}\right)\;...\;A_{x}\left(t_{k+n-1}\right)\right]$ cropped by the time window $\left[t_{k}\;\;\;t_{k+n-1}\right]$ is normalized. The original data sequence ($A_{x}$) is subtracted and divided by its own average ($\mu_{x}$) and standard deviation ($s_{x}$) and the normalized data sequence ($A_{x}^{\prime }$) is saved for next process. The normalization process was applied in the same way to the y-axis and z-axis acceleration data.

(1) $$\begin{array}{c} \mu_{x}=\sum_{i=k}^{k+n-1}A_{x}\left(t_{i}\right)/n,\;\;s_{x}=\sqrt{\sum_{i=k}^{k+n-1}{\left(A_{x}\left(t_{i}\right)-\mu_{x}\right)}^{2})/n},\\ A_{x}^{\prime }\left(t_{i}\right)=\left(A_{x}\left(t_{i}\right)-\mu_{x}\right)/s_{x}\;\;\;\;,\;\;i=k,k+1,\ldots k+n-1. \end{array}$$

**Figure 3:**
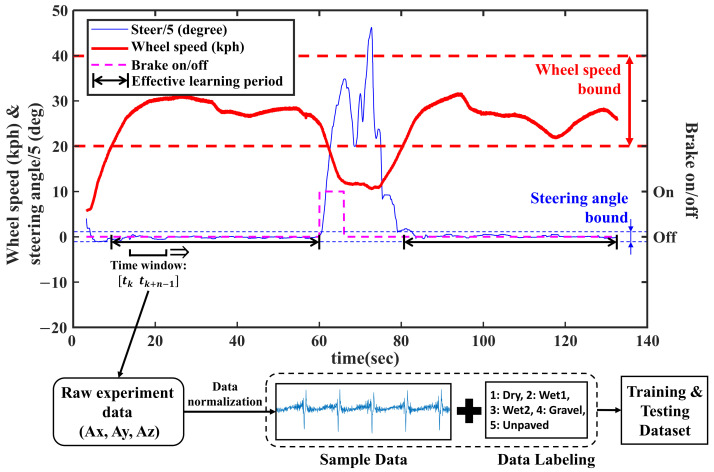
Dataset preparation process for deep learning.

After normalizing the data, each sample data was labeled with the types of road surfaces (1: dry asphalt, 2: wet asphalt 1, 3: wet asphalt 2, 4: gravel, 5: unpaved) and stacked on the training or testing dataset. Figure 3 shows the process of obtaining the training and testing dataset from the experimental results.

Through this process, a training dataset with 100,000 samples (20,000 samples per road surface) and a testing dataset with 20,000 samples (4000 samples per road surface) were acquired for each x-, y- and z-axis acceleration sensor signal. The training and testing datasets were acquired from independent effective learning data periods to prevent the network from learning the testing dataset in advance.

### 2.4. Design of FCNN and CNN Structure

Intelligent tire sensor datasets for each road surface type are trained with two types of DNNs: a fully connected neural network (FCNN) and a 1-dimensional convolutional neural network (CNN).

**Figure 4:**
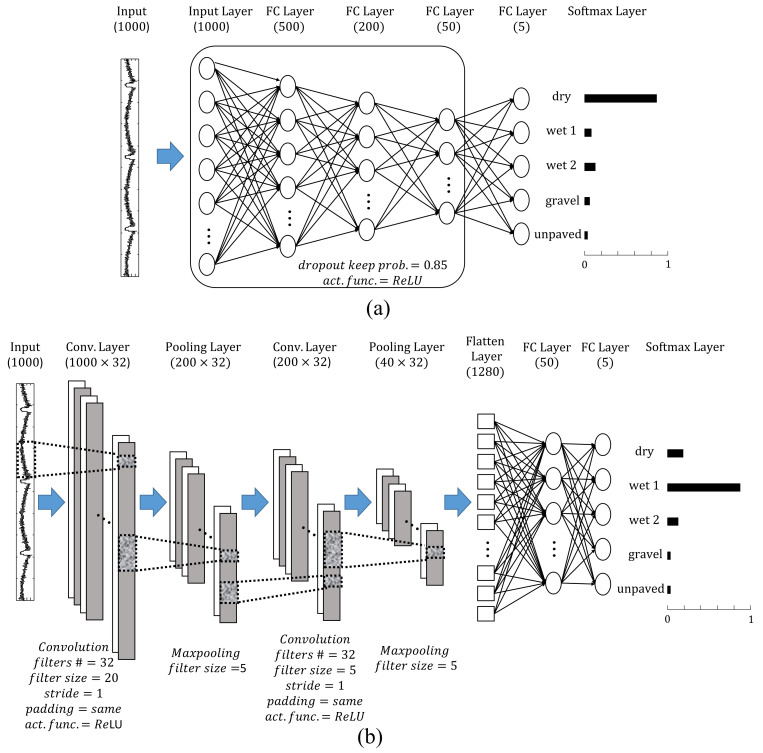
Structure and parameters of each layer of the (**a**) FCNN and (**b**) 1-D CNN.

An FCNN is a basic structure of an artificial neural network consisting of a series of fully connected layers that connect every node in each layer to every node in the next layer and finds a nonlinear relationship between input and output data by optimizing the internal parameters of dense networks [29]. The major characteristic of FCNNs is that they are “structure agnostic”; that is, they assign equal importance to each value of input sequence data regardless of position in the sequence. In this study, random dropout (dropping some proportion of the nodes during training) with a certain probability is applied to each hidden layer of the FCNN to prevent overfitting of the learning network. At the end of the FCNN, a softmax layer is placed so that the output of the network is suitable for classification. Figure 4a shows the schematic of the FCNN.

A CNN is specialized kind of DNN architecture that can be used to train spectral variations and model spectral correlations existing in the signals and is most commonly applied to learning images [30]. Typical CNNs consist of sequence of three main layers—convolution and pooling and fully-connected layers [29]. Figure 4b shows the schematic of a typical 1-dimensional CNN. The convolutional layer is a special form of network that can extract the spatial features from images (or sequential features from 1-dimensional data) by training the filters (kernels) inside the layer so that the CNN can identify the patterns or objects. The pooling layer receives the output data from the convolution layer as the input and is used to reduce the size of the output data or to highlight specific data. After sufficiently recognizing the features of the image or sequential data with the convolutional layer, the output of the last pooling layer is flattened and connected to the fully connected layer and the softmax layer to finally output the classification result.

## 3. Results and Discussion

### 3.1. Network Training Process

For two types of training networks (FCNN and CNN), the training was repeated by changing the structure and parameters of the network until the highest average accuracy from the training results with x-, y- and z-axis acceleration datasets ($A_{x}$, $A_{y}$ and $A_{z}$ datasets) was found. Each network was trained based on the adaptive moment estimation (Adam) optimizer, a batch size of 64 and a learning rate of 0.01. To compare the performances of the two networks, the time window length was fixed to 1000; that is, the input data for training corresponded to a 1 s duration in the experiment. Of the training dataset, 20% was separated as a validation set to check overfitting and to early-stop the training process. If, during training, there is a mismatch between the values of the loss function in the training set and the validation set, training stops before the network overfits.

To find the optimal network with the highest accuracy, the same dataset was repeatedly trained by FCNNs and CNNs by changing the number of total layers, number of nodes in fully-connected layer, number of filter and filter size of convolution layer, and filter size of maxpooling layers. A network with a too-complex structure overfits the training dataset, reducing the accuracy of the test dataset, and a network with a too simple structure cannot acquire all the information in the training dataset, so it is important to find a network with an appropriate complexity.

**Figure 5:**
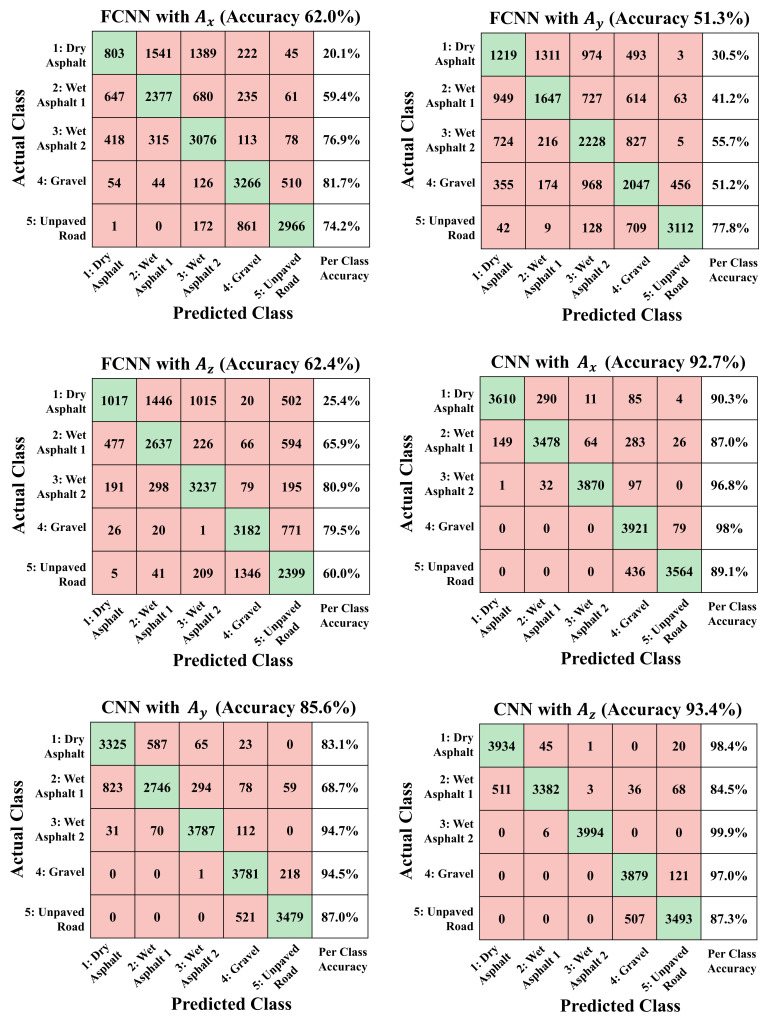
Confusion matrix of 5-class classification results from FCNN and CNN with x-, y- and z-axis acceleration test datasets.

The final structure and parameters of each network are expressed in Figure 4, and the performance of trained networks with different training datasets are expressed with the confusion matrix results with the test dataset as shown in Figure 5.

The accuracy of the network was calculated as the proportion of cases where predicted class by the network and actual class matched among the entire test dataset.

### 3.2. Training Results of DNNs Using Acceleration Dataset of Each Axis

The training results in Figure 5 show that the trained FCNN achieved 5-class classification accuracies of 62.0%, 51.3% and 62.4% for the $A_{x}$, $A_{y}$, and $A_{z}$ test datasets, respectively and the accuracies of the learning results show tat the FCNNs is insufficient to be used as a road condition classifier regardless of the type of acceleration sensor signal.

**Figure A2:**
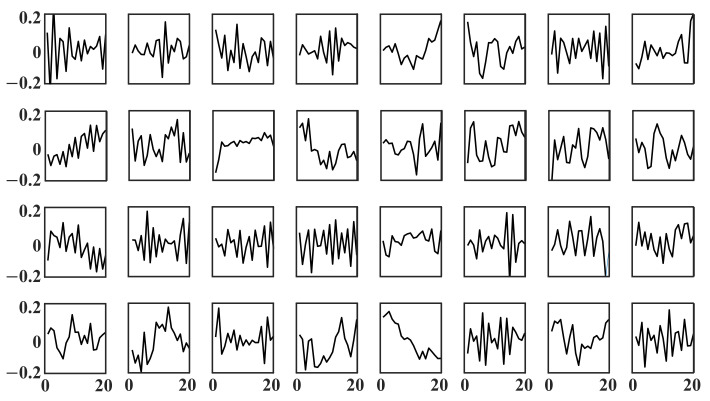
Filters of the First convolutional layer of the trained CNN based on x-axis acceleration signal dataset.

**Figure A3:**
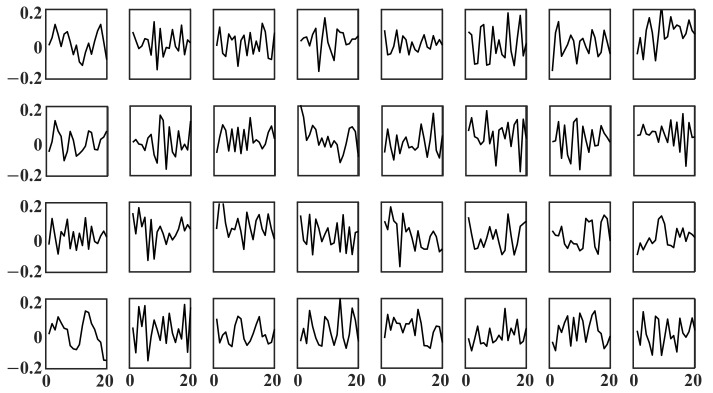
Filters of the First convolutional layer of the trained CNN based on y-axis acceleration signal dataset.

**Figure A4:**
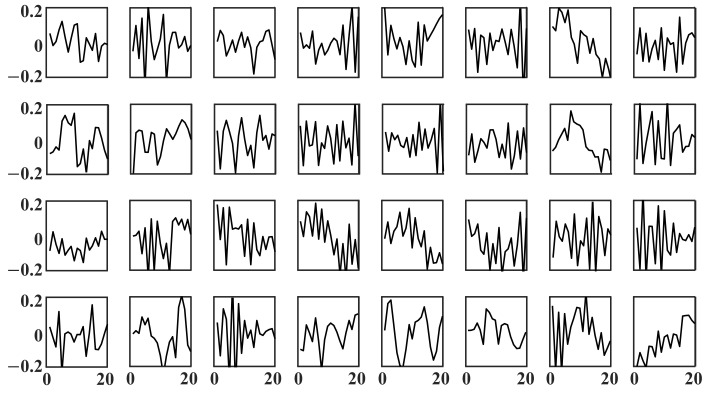
Filters of the First convolutional layer of the trained CNN based on z-axis acceleration signal dataset.

In contrast, the training results in Figure 5 show that the 1-D CNN achieved 5-class classification accuracies of 92.7%, 85.6% and 93.4% for the $A_{x}$, $A_{y}$, and $A_{z}$ test datasets, respectively. The learning result shows that the x-axis and z-axis acceleration sensor data have sufficient information on the type of road surface, and the 1-D CNN architecture can extract the features from the sequential data and secure a meaningful level of accuracy as a road surface condition classifier. The filters of the first layer of the learned CNN network shown in Figure A2, Figure A3 and Figure A4 reveal which features the network considered important from the training datasets.

Examining the learning result of the CNN in more detail for each class reveals that the accuracy of the learned network varies depending on road surface class. For example, for the CNN training result based on the $A_{x}$ dataset, classes 3 and 4 have very high classification accuracies, which are above 95%, but the remaining classes have accuracies of 87–90%, which are lower than the overall accuracy of the network. One of the reasons for decreasing the accuracy of the CNN-based learning network is the low classification accuracy of wet asphalt with 1 mm waterfilm regardless of the type of dataset. In particular, most of the confusion from wet asphalt with 1 mm waterfilm test data occurs for dry asphalt. Therefore, because the waveforms of the two classes are difficult to distinguish clearly even for humans as shown in Figure A1, there is a limitation in learning different feature points between these waveforms even in the learning process based on the CNN. Another factor in the reduction of overall training accuracy is the confusion between gravel and unpaved road. Networks can clearly distinguished gravel and unpaved road from dry asphalt or wet asphalt since the waveform of accelerometer signals from gravel and unpaved road classes differ from those of other classes, as shown in Figure A1. However, results show that the trained CNN has limitations in perfectly learning the features that differentiate gravel and unpaved road. If the number of layers, filters, or nodes of the network is increased than the CNN structure in Figure 4 in order for the network to learn more information, the learning accuracy for the training dataset increases, but network overfitting occurs, which in turn degrades the performance of the test dataset.

Figure 5 shows that the training results from $A_{y}$ has lower accuracy than the training result from $A_{x}$ and $A_{z}$ because the tire of a straight driving vehicle has less interaction with the road surface in the lateral direction than in the longitudinal or vertical directions. However, except for class 2, the trained network can classify with a meaningful level of accuracy (over 80%) and especially for classes 3 and 4, an accuracy of over 94% is obtained. This result shows that even for straight driving, the $A_{y}$ signal has information that can distinguish the road surface type, and some studies also confirm this result [31]. As seen in Figure A1, although the waveform of $A_{y}$ does not have distinct waveform characteristics for each road surface compared to $A_{x}$ or $A_{z}$, it can be distinguished to some extent with respect to the size and frequency of the signal.

Although fully connected networks can be applied very widely due to their agnostic nature, FCNN tend to perform worse than special-purpose networks, such as CNN that extract patterns from sequential data in this case. Therefore, it is difficult to improve the accuracy of FCNN beyond a certain level, while trained CNN achieves higher accuracy by identifying important signal features that occur during the contact period for each type of road surface.

Not only the type of network and dataset but also the normalization process of the training dataset greatly affects the performance of the overall learning result. If the CNN is trained using $A_{x}$ and $A_{z}$ training datasets without normalization process while maintaining the network architectures and hyperparameters, the accuracies of the trained CNNs drop to 64.1% and 61.9%, respectively. These results indicate that the variations in amplitude and offset value in the acceleration signals due to changes in vehicle speed degrade the quality of the training data and the normalization process can eliminate such negative effects.

**Figure 6:**
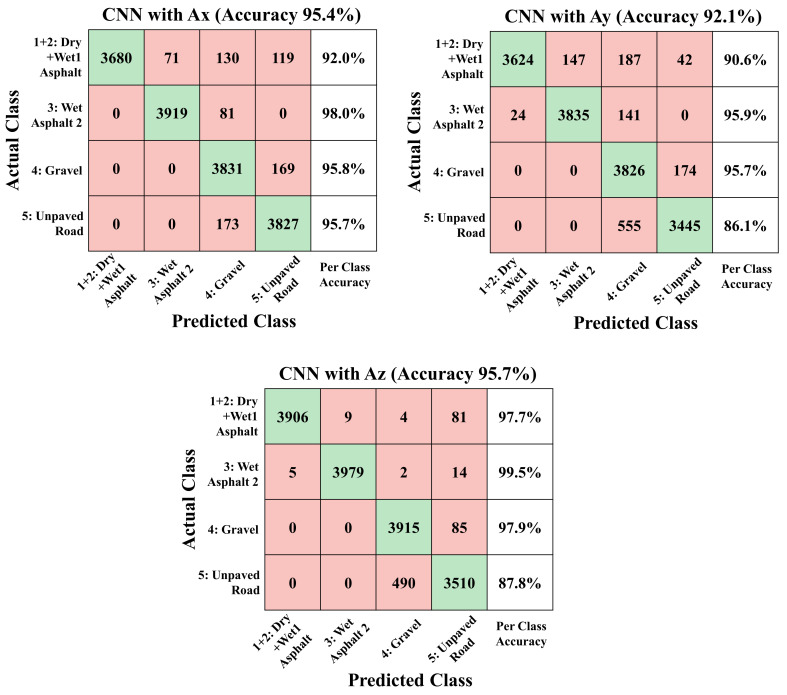
Confusion matrix of 4-class classification results from CNN with x-, y- and z-axis acceleration test dataset.

As a result of training the same CNN structure on four classes by combining dry asphalt and wet asphalt with 1mm waterfilm, which are the classes with many confusions at 5 class classification, trained network have accuracy of 95.7%, 86.1%, and 97.9% for $A_{x}$, $A_{y}$, and $A_{z}$ dataset as shown in Figure 6. The overall accuracy of the trained networks is clearly improved because the classes that have many confusions are combined into one class and the total number of classes has decreased. In addition, confusion between gravel and unpaved roads is also reduced by combining classes 1 and 2, apparently because networks do not need to train the difference between classes 1 and 2, so the network can allocate more capacity to distinguishing the rest of the classes.

As much as selecting a network with an appropriate structure, it is important to correctly determine the number of classes and the type of dataset according to the purpose of use. If the road classification result is used in the vehicle chassis stability control logic used in extreme conditions, such as heavy rain and off-road conditions, it is desirable to have high accuracy for roads under these conditions and it would be more proper to have a 4-class classification strategy, as shown in Figure 6. In addition, to ensure that per class accuracy for all classes is guaranteed more than 90%, the $A_{x}$ rather than the $A_{y}$ or $A_{z}$ dataset can be chosen as an input signal. Moreover, the gravel and unpaved road can be combined into one off-road class and the network is trained with three classes for higher accuracy.

### 3.3. Training Results of CNNs Using Multi-Axis Acceleration Dataset

**Figure 7:**
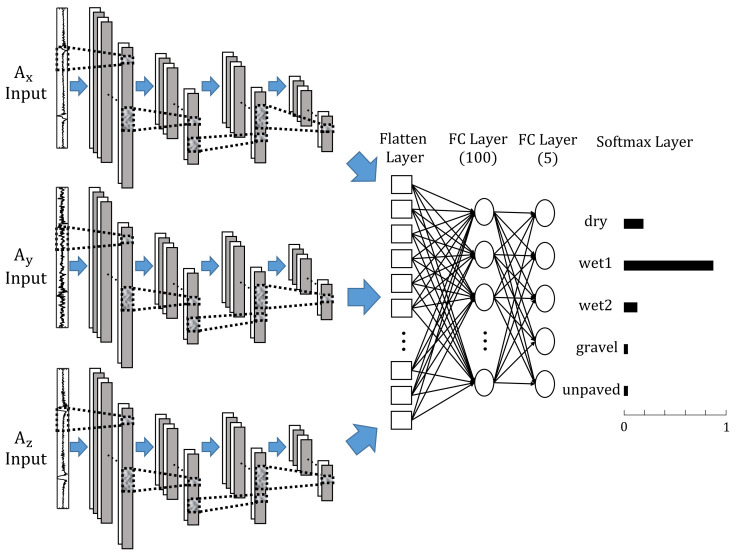
Structure and parameters of each layer of modified 1-D convolutional neural network with multiple input.

If a network is trained with all the axes signals from the intelligent tire sensor at the same time, the performance of the road condition classification network can be improved from the networks using only single axis signals. By copying the layer block from the first convolutional layer to the second pooling layer of Network in Figure 4b and tying them into a flatten layer, a network that receives a number of sequential data as input and gives the 5-class lassification result as an output is designed as shown in Figure 7. The newly designed network trains pattern characteristics independently for each of two or three sequential data inputs, and the pattern recognition results through the each convolution layer block for each input are collected to derive a final classification result through fully-connected and softmax layers.

**Figure 8:**
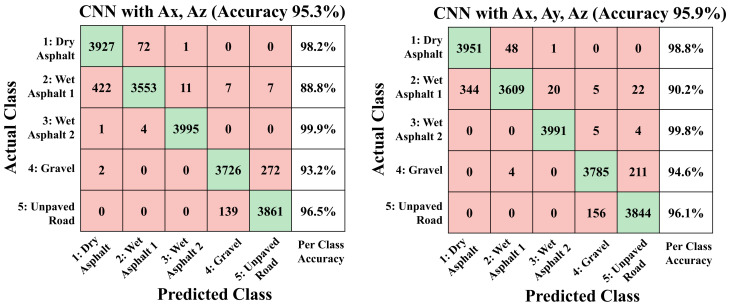
Confusion matrix of 5-class classification results from CNN with (x-axis + z-axis) and (x-axis + y-axis + z-axis) acceleration test dataset.

Figure 8 shows the performance of the multiple-input network trained by ($A_{x}$, $A_{z}$) dataset and ($A_{x}$, $A_{y}$, $A_{z}$) dataset in the form of a confusion matrix. The trained networks using the multi-axis acceleration dataset have accuracies of 95.3% and 95.9% for ($A_{x}$, $A_{z}$) and ($A_{x}$, $A_{y}$, $A_{z}$) test dataset, respectively, and is improved from the networks trained by single-axis acceleration signal in Figure 5. Even when comparing the accuracy of each class, it can be seen that the per-class accuracy of the multiple-input network has improved for all classes. The results show that the performance of the proposed multiple-input networks properly combine the pattern information from acceleration signals of each axis. In addition, as the training result using the ($A_{x}$, $A_{y}$, $A_{z}$) dataset has a slightly higher accuracy than the training result using the ($A_{x}$, $A_{z}$) dataset, it can be seen that waveform pattern information acquired from the $A_{y}$ signal also has contribution to the classification of road conditions.

### 3.4. Applying Road Condition Classification Network in Time Domain

From a real-time application point of view, the proposed road condition classification algorithm has a practical advantage in that it enables estimating the road surface condition at any time instance regardless of the position of the wheel, while previous studies [25,27] require a wheel position sensor or an algorithm that can cut the signals by each wheel rotation to extract the exact contact period of intelligent tire sensor with road surface. In addition, applying the trained network to actual experimental data similarly to real-time application verified that the trained network takes an operation time of less than 3 ms to classify the road condition. Because the road condition can be estimated at any time with fast enough sampling time, the proposed algorithm has sufficient performance to be utilized in vehicle controllers as a real-time road surface classifier.

**Figure 9:**
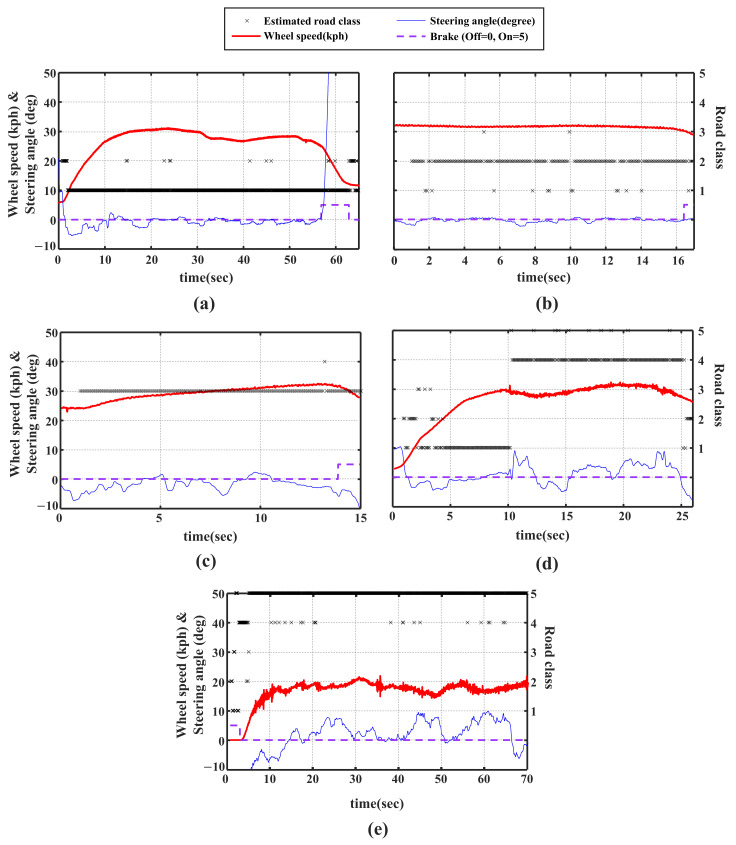
Result of applying road classification network to driving experiment data at (**a**) dry asphalt road, (**b**) wet asphalt with 1 mm waterfilm, (**c**) wet asphalt with 4 mm waterfilm, (**d**) gravel, and (**e**) unpaved road.

The Figure 9 shows the result of applying the 5-class classification CNN with multi-axis acceleration input ($A_{x}$, $A_{y}$ and $A_{z}$) in Figure 8 to driving experimental data for each road condition, and the result is the same as the result of applying the road classification algorithm in real time. Several characteristics of the developed classification algorithm are shown by the results. First of all, there is a 1 s delay at the beginning of all experiments because sequential data corresponding to 1 s are required as an input to the network. In addition, as seen from the Figure 9a,d,e, since the learning was performed for 10–70 kph, the estimation is not accurate when the vehicle speed is less than 10 kph. Last, as shown in Figure 9d, the road classification algorithm allows us to clearly identify the transition from dry asphalt to gravel in 10 s.

### 3.5. Trade-Off between Length of Time Window and Accuracy of Network

**Figure 10:**
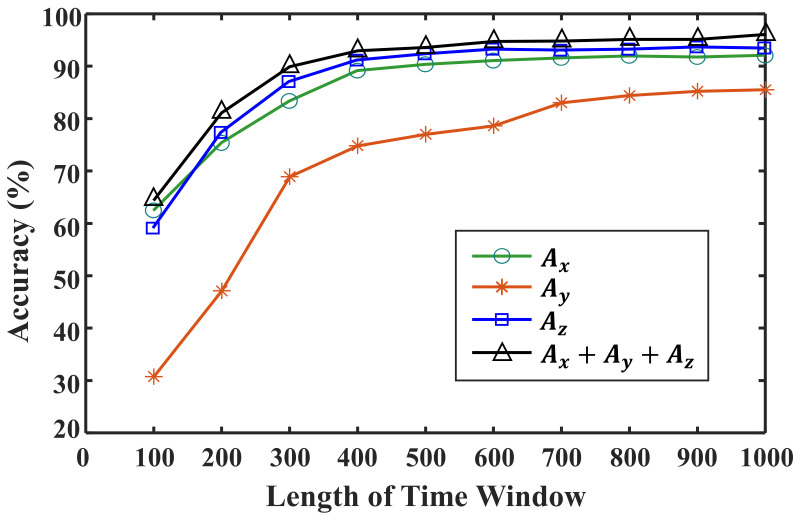
Accuracy analysis under different time window lengths.

Finally, we analyzed the effect of the time window length on the performance of the CNN network. The learning results shown earlier were acquired using a time window length of 1000. Therefore, if this network were to be applied to estimate the condition of the road surface in real time, a 1 s delay would occur. Because a 1 s delay in road surface condition estimation would reduce the control or notification performance, the delay should be reduced as much as possible. Therefore, a CNN model with the same structure and parameters is trained while steadily reducing the time window size. Figure 10 shows the results of training after reducing the length of the CNN’s input data from 1000 to 100. The results show that the model accuracy decreases as the time window size is reduced and the CNN cannot guarantee the learning results with fewer than 400 input data points.

As a result, it will be the most optimal network to adopt the shortest time window length that satisfies the minimum accuracy required for the network referring to Figure 10. For example, if minimum requirement of overall accuracy for a road classification network is 90%, the time window length of 400 is the most optimal choice and, if an accuracy of 95% or more is required, the time window length of 1000 is optimal.

## 4. Conclusions

In this study, a database containing tire–road interaction information in contact patches was collected on various road surfaces by using an intelligent tire system with an acceleration sensor attached to the inside of the tire. Based on this database, two types of deep learning algorithms are trained and their performance is compared to classify the road surface type. The results showed that a 1-dimensional CNN is more suitable than a FCNN for extracting road surface identification information from acceleration sensor signals. The results also indicate that x- and z-axis acceleration signals contain more information on the type of road surface than the y-axis acceleration signal, and the learning result based on the z-axis acceleration dataset has the highest overall accuracy. Additionally, by analyzing how the accuracy of the trained network is affected by the number of classes and the length of the time window, a criterion for selecting an appropriate network was suggested.

Compared with the existing research on road surface classification, the proposed algorithm is more practical because it can provide road surface conditions continuously and fast enough to be utilized in the chassis controller of a vehicle. Moreover, due to the characteristics of the DNN, the developed road classification network can improve its performance by using an additional experimental dataset. For example, for a rapid acceleration period where the accuracy of road surface estimation is currently inferior, the performance can be improved by additional learning with the experimental datasets for the acceleration driving condition. In the same way, by securing additional datasets for various vehicle conditions such as tire type, tire pressure, vehicle load, and various driving conditions, the road classification network can be improved so that it can operate robustly in all situations.

The currently developed algorithm classifies the road surface condition with respect to the acceleration sensor signal for only 1 axis but future research will be conducted to improve the accuracy and usability of the road surface classification by using the 3-axis acceleration sensor signal at the same time or by using the driving condition, vehicle body acceleration and gyro signal.

## Abbreviations

The following abbreviations are used in this manuscript:

ADAS	Advanced Driving Assistance System
DNN	Deep Neural Network
FCNN	Fully Connected Neural Network
CNN	Convolutional Neural Network
DAQ	Data Acquisition
